# A Cytokine–Cytokine Interaction in the Assembly of Higher-Order Structure and Activation of the Interleukine-3:Receptor Complex

**DOI:** 10.1371/journal.pone.0005188

**Published:** 2009-04-07

**Authors:** Raja Dey, Kunmei Ji, Zhigang Liu, Lin Chen

**Affiliations:** 1 Department of Biological Sciences, Molecular and Computational Biology, University of Southern California, Los Angeles, California, United States of America; 2 Institute of Allergy and Immunology, School of Medicine, Shenzhen University, Shenzhen, Guangdong, China; University of Washington, United States of America

## Abstract

Interleukine-3 (IL-3) binds its receptor and initiates a cascade of signaling processes that regulate the proliferation and differentiation of hematopoietic cells. To understand the detailed mechanisms of IL-3 induced receptor activation, we generated a homology model of the IL-3:receptor complex based on the closely related crystal structure of the GM-CSF:receptor complex. Model-predicted interactions between IL-3 and its receptor are in excellent agreement with mutagenesis data, which validate the model and establish a detailed view of IL-3:receptor interaction. The homology structure reveals an IL-3:IL-3 interaction interface in a higher-order complex modeled after the dodecamer of the GM-CSF:receptor complex wherein an analogous GM-CSF:GM-CSF interface is also identified. This interface is mediated by a proline-rich hydrophobic motif (PPLPLL) of the AA′ loop that is highly exposed in the structure of isolated IL-3. Various experimental data suggest that this motif is required for IL-3 function through receptor-binding independent mechanisms. These observations are consistent with structure-function studies of the GM-CSF:receptor complex showing that formation of the higher-order cytokine:receptor complex is required for signaling. However, a key question not answered from previous studies is how cytokine binding facilitates the assembly of the higher-order complex. Our studies here reveal a potential cytokine–cytokine interaction that participates in the assembly of the dodecamer complex, thus linking cytokine binding to receptor activation.

## Introduction

IL-3 and the closely related short-chain cytokine GM-CSF and IL-5 are a subclass of the cytokines/growth factors secreted largely by activated T cells [1]. These cytokines regulate the survival, proliferation and differentiation of hematopoietic cells, while GM-CSF has also been shown to regulate the function of dentritic cell and T cells [2], [3]. Dysfunction of this family of cytokines has been implicated in a variety of pathologies including rheumatoid arthritis and leukemia [4]–[8], whereas treatment with recombinant cytokine of this family showed beneficial effect in a number of clinical conditions [9]–[12], making them important targets of therapeutic development.

IL-3, GM-CSF and IL-5 function by binding to their cognate receptors expressed on the surface of cells in the hematopoietic system. The receptors for IL-3, GM-CSF and IL-5 consist of a cytokine-specific alpha subunit and a common beta subunit (βc) that are both required for function [13]–[17]. The mechanism by which cytokine binds and activates its receptor has been the subject of extensive structural and functional studies [18]. As first revealed in the human growth hormone receptor complex structure [19], a general theme from these studies is that the binding of cytokine induces dimerization of receptor subunits, which brings signaling molecules (e.g. kinases) associated with the cytoplasmic region of receptor subunits in close proximity for trans-activation. However, higher-order cytokine:receptor complexes have been implicated in receptor-mediated signaling [20], and it becomes increasingly clear that each cytokine:receptor pair has unique features in binding and activation mechanism. Comparison of related cytokine:receptor complexes have been a powerful way to explore the mechanisms of cytokine signaling [20]–[23].

The binding of IL-3 to its receptor has been investigated extensively using various approaches, including constructing interspecies chimera [24], [25], mapping the epitopes of neutralizing antibodies [24], [26], and mutagenesis [26]–[32]. These studies provided rich biochemical and functional data on sites of IL-3 that are important for receptor binding and signaling activation. A model of IL-3:receptor complex was also generated based on the human growth hormone hGH:receptor complex structure [19], [33]. While this model provided satisfactory interpretations for some of the mutagenesis data, its accuracy is limited by the low homology between the IL3:receptor complex and the hGH:receptor complex. The crystal structure of a GM-CSF:receptor complex has recently been determined [34]. IL-3 has a higher homology to GM-CSF than hGH. Moreover, the receptors for IL-3 and GM-CSF share a common beta subunit. Thus the structure of the GM-CSF:receptor complex offers a much better template for building a more accurate model of the IL-3:receptor complex.

The mechanism by which IL-3, GM-CSF and IL-5 activate their receptors has been a longstanding puzzle. Although the cytoplasmic domain of the receptor alpha subunit is required for function, the signaling kinase (JAK2) seems to bind only the beta subunit that adopts an extended structure. Thus the classical model of cytokine-induced dimerization of receptor subunits cannot explain signal activation for this family of cytokines. Although it remains controversial about which JAK kinases associate with the receptor subunits and whether signaling is initiated by homo- or hetero dimerization, the crystal structure of the GM-CSF:receptor complex reveals a dodecameric assembly that provides a plausible mechanism for receptor activation wherein the tyrosine kinase JAK2 associated with the beta subunit are brought together for trans phosphorylation [15]–[17]. Mutations on the receptor beta subunit designed to disrupt the dodecamer assembly interface diminished the activity of the receptor, providing functional evidence that the higher-order GM-CSF:receptor complex is required for signaling [34].

The dodecameric assembly of cytokine:receptor complex has also been proposed as part of the receptor activation mechanism for IL-3 and IL-5 [34]. Indeed, beta subunits containing mutations that presumably disrupt dodecamer assembly showed decreased ability to mediate IL-3-dependent signaling [34]. However, an intriguing question about this model of receptor activation is the role of the cytokine itself. The protein interface in the GM-CSF:receptor dodecamer shown to be functionally important by mutagenesis is mediated entirely by the beta subunit. How the cytokine itself controls the formation of the dodecamer complex and signaling activation is not clear.

To address this question, we generated a homology model of the IL-3:receptor complex based on the crystal structure of the GM-CSF:receptor complex and took advantage of the vast amount of biochemical data on the IL-3:receptor complex. The excellent agreement between the model and previously published mutagenesis data suggest that the homology structure represents a reliable model for IL-3:receptor interaction. We identified a cytokine-cytokine interaction interface in the modeled structure of the IL-3:receptor complex and in the crystal structure of the GM-CSF:receptor complex that participates in the assembly of the dodecamer. In IL-3 this interface is mediated by a proline-rich and exposed hydrophobic motif (PPLPLL) in the AA′ loop. Some modifications of this motif by mutagenesis and by the binding of neutralizing antibodies have been shown to affect the activity of IL-3 without significantly changing its binding affinity for the receptor [35]. These observations are in agreement with mutagenesis studies of the GM-CSF:receptor dodecamer showing that high affinity binding of cytokine to the receptor is not sufficient for activation and that the formation of the higher-order complex is an obligate step to initiate signaling. Our findings not only provide further support for the dodecamer model of receptor activation in the IL-3:receptor complex, but more importantly, reveal a cytokine-cytokine interaction interface that participates in the assembly of the dodecamer and hence signal initiation. The structural model generated by this study will serve as a basis for further studying the functional mechanisms of the IL-3:receptor complex and for the development of therapeutics.

## Materials and Methods

### Selection of templates

The crystal structures of the GM-CSF:receptor complex (3.3 Å resolution) [34] and the IL-4:IL-4R:IL-13RA ternary complex (3.02 Å resolution) [23]were used as templates to develop a homology model of human IL-3 (hIL-3) bound to its receptor (the hIL-3:receptor complex). The coordinates for both structures were taken from the Brookhaven Protein Data Bank using the accession numbers 3CXE and 3BPN, respectively. The NMR structure of a functionally active hIL-3 variant SC-65369 (PDB accession number 1JLI) was also taken from the Protein Data Bank to build a structural model for the wild type hIL-3 (14–125). The amino acid sequence of hIL-3 receptor α-chain was obtained from ExPASy Proteomics Server (protein accession number P26951). To select templates for modeling the IL-3 receptor α-chain, we searched for its homologous sequences using BLAST (http://www.ncbi.nlm.nih.gov/blast) against PDB database. It turns out that the two domains of the IL-3 receptor α-chain align optimally to two different protein sequences. The best template (with the highest sequence homology) for domain 1 of IL-3 receptor α-chain is chain C of 3BPN.pdb, whereas that for domain 2 is chain C of 3CXE.pdb.

### Homology modeling

Homology models of hIL-3 receptor α-chain domain 1 (Pro103-Ile207) and domain 2 (Leu208-Glu292) were developed based on the crystal structure of IL-13 receptor α-chain (IL-13RA) and GM-CSF receptor (GMR) α-chain domain 2 respectively, using the program MODELLER [36]. The corresponding sequence alignments are shown in Figure S1 and Figure S2. Domain 1 of GMR α-chain is poorly defined in the crystal structure of the GM-CSF:receptor complex[34]. To get the relative inter-domain orientation, a combined model of hIL-3 receptor α-chain (including both domain 1 and 2) was also developed based on IL-13RA (sequence alignment is shown in Figure S3). Because domain 1 and domain 2 of IL-3 receptor α-chain were modeled on different templates, the linker region (residues Phe202 to Pro210) was rebuilt with plausible backbone conformation and side chain rotamers using program O [37]. The wild type hIL-3 model was generated by in silico mutations from its mutant NMR structure using the program O. A total of 14 substitutions were introduced in the IL-3 NMR structure. The side chains of newly introduced amino acid residues were manually adjusted to remove local clashes and to allow potential hydrogen bonding and electrostatic interactions. All the models were then put through energy minimization in MODELLER with fixed backbones.

### The hIL-3:receptor complex model

**Figure 1:**
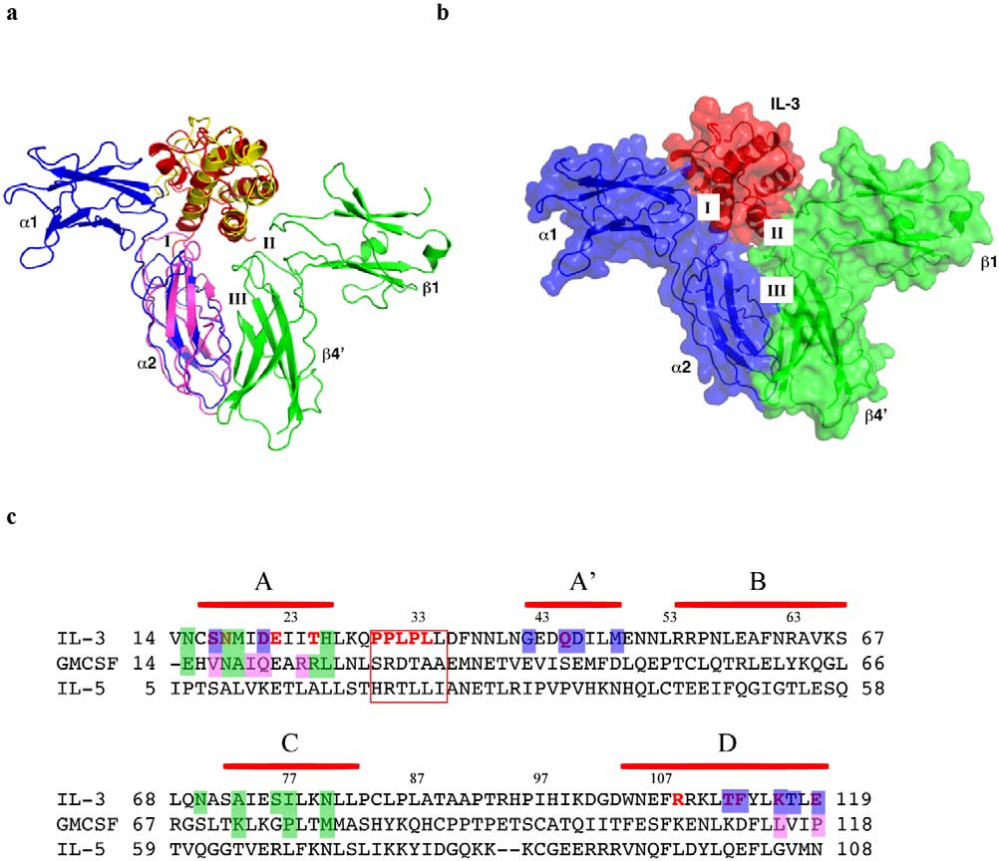
Modeling of the IL-3:receptor complex. (A) The structure of IL-3 (red) is superimposed on that of GM-CSF (yellow). The IL-3 receptor alpha chain domain 2 (α2, blue) is superimposed on the corresponding domain of GM-CSF receptor alpha chain (light magenta). The position of IL-3 receptor alpha chain domain 1 (α1, blue) is fitted as described in text. Domain 1 of one beta subunit (β1) and domain 4 of the other (β4′) are shown in green. The rest of the beta dimer is not shown. The three interfaces of the complex discussed in the text are labeled. The color scheme is used throughout the illustration unless otherwise indicated; (B) The resulting model of the IL-3:receptor complex after removing GM-CSF and its receptor alpha subunit, shown in ribbon covered with transparent surface; (c)sequence alignment of IL-3, GM-CSF and IL-5. Helices in IL-3 are indicated by horizontal bar in red. Residues discussed in the text are highlighted in different colors: green shade, residues interacting with the receptor β-subunit in IL-3 and GM-CSF; blue shade, residues of IL-3 that interact with the receptor α-subunit; magenta shade, residues of GM-CSF that interact with the receptor α-subunit; red font, functionally important residues from mutagenesis studies. Boxed residues are involved interface V interaction. Residue numbering above the sequence is for IL-3.

The structure of isolated GM-CSF is similar to its receptor bound form [34], [38]. We assume that the structure of hIL-3 bound to its receptor is also similar to its free from. We therefore docked IL-3 as a rigid body on the GM-CSF:receptor complex by superimposing its structure with that of GM-CSF (Figure 1A). The superposition is based on the backbone of the four conserved helices (A–D) that shows an RMSD of 2.7 Å for 50 C-α atoms (Figure S4). The homology model of hIL-3 receptor α-chain is docked onto the GM-CSF:receptor complex by superimposing its domain 2 with that of GM-CSF receptor α-chains (Figure S5A). Domain 1 of hIL-3 receptor α-chain, which is constructed based on the template of IL-13RA domain1 (Figure S5), is fitted into the ternary complex based on the inter-domain orientation of IL-13RA with minor manual adjustments (see below). GM-CSF and its receptor alpha chain were then removed from the combined coordinates. With the remaining common β-chain, hIL-3 and the receptor alpha chain were therefore placed together using the molecular framework defined by the crystal structure of the GM-CSF:receptor complex (Figure 1B). The complex model was subject to energy minimization with fixed backbone using MODELLER. A few local clashes in the final model were removed manually in O by choosing different side chain rotamers. Throughout the modeling process, only domain 1 and domain 4 from two different hIL-3 receptor β-subunits that are in contact with IL-3 were included in the model refinement procedures. A multiple sequence alignment among IL-3, GM-CSF, and IL-5 is shown in Figure 1C with residues discussed in the text highlighted. Overall, the homology model is based on the identical beta subunit and closely related cytokine structures between IL-3 and GM-CSF. Although the accuracy of the detailed hIL-3 receptor α-chain structure may be limited by the modest sequence identity, the overall arrangement of its two domains in the final complex can be assigned with high confidence, so the role of the receptor α-chain in the assembly of the dodecamer complex can be assessed with reasonable certainty (see below).

## Results

### Overall structure of the IL-3:receptor complex

#### The canonical mode of cytokine/receptor interaction

IL-3 is bound to the receptor through interfaces with the alpha subunit (Interface I) and beta subunit (interface II). Upon binding to their cytokine ligand, the alpha and beta subunit also interact with each other through a binding interface termed interface III (Figure 1A and 1B). The buried solvent accessible surface area between IL-3 and its receptor is 2267 Å^2^, which is comparable to that of the GM-CSF:receptor complex. Domain 2 of IL-3Rα shows notable difference from the corresponding domain of GMRα (Figure 1A). Although most of the residues inside the beta sandwich core are conserved (Figure S5A), the loop region (residues Gln241–Gln247) involved in binding to the beta subunit and several other loops (residues Arg229-Lys235 and residues Arg275-Phe281, here residues are numbered according to hIL-3Rα) show significant divergence from GMRα. These loops of IL-3Rα are substantially shorter than their counter parts in GMRα (Figure 1A, Figure S5A), their structure and trajectory are highly constrained by the beta sandwich core. Thus, the roles of these loops in binding with the beta subunit and the cytokine can be assessed with reasonable certainty. Domain 1 of IL-3Rα also likely has some differences from the corresponding domain of GMRα. In the crystal structure of the GM-CSF:receptor complex, domain 1 of GMRα is poorly defined, suggesting its relatively high flexibility in the complex. In the homology model of the IL-3:receptor complex, the placement of hIL-3Rα domain 1 is guided by the inter domain orientation of IL-13Rα The final position of hIL-3Rα domain 1 is also slightly adjusted as a rigid body to account for potential IL-3:IL-3Rα interactions defined by mutagenesis studies (see below).

#### Higher-order structure of the IL-3:receptor complex

**Figure 2:**
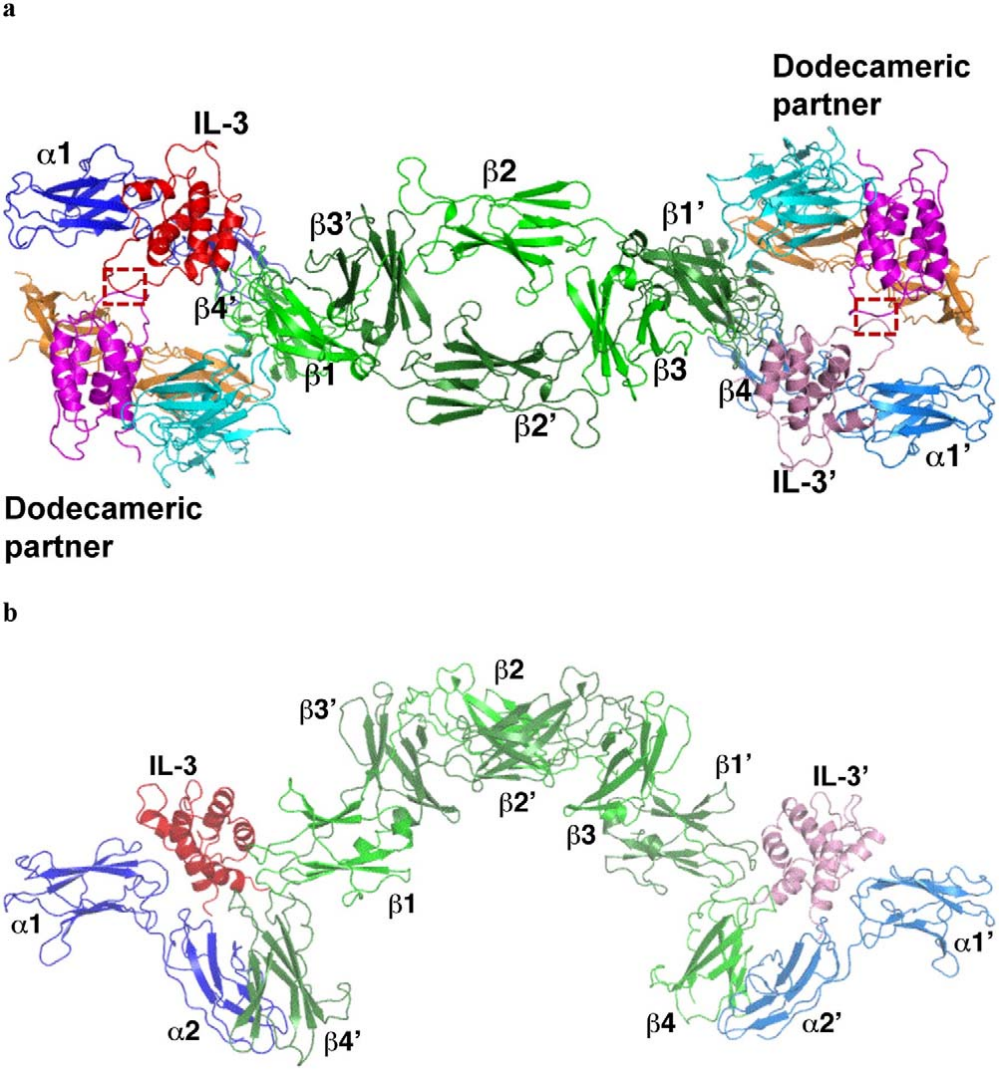
Higher-order structure of the IL-3:receptor complex. (A) Assembly of one trimeric IL-3 (red), IL-3Rα (blue), and IL-3Rβ (green) cytokine/receptor complex with the other (pink, marine and forest) into the hexamer. The dodecameric partners at each end are shown in magenta (IL-3), orange (IL-3Rβ) and cyan (IL-3Rα, domain 1 is omitted in this view), with the interaction interface indicated by a dashed box. (B) Side view of the complex showing the arched structure. Only the foreground structure is shown in this view.

**Figure 3:**
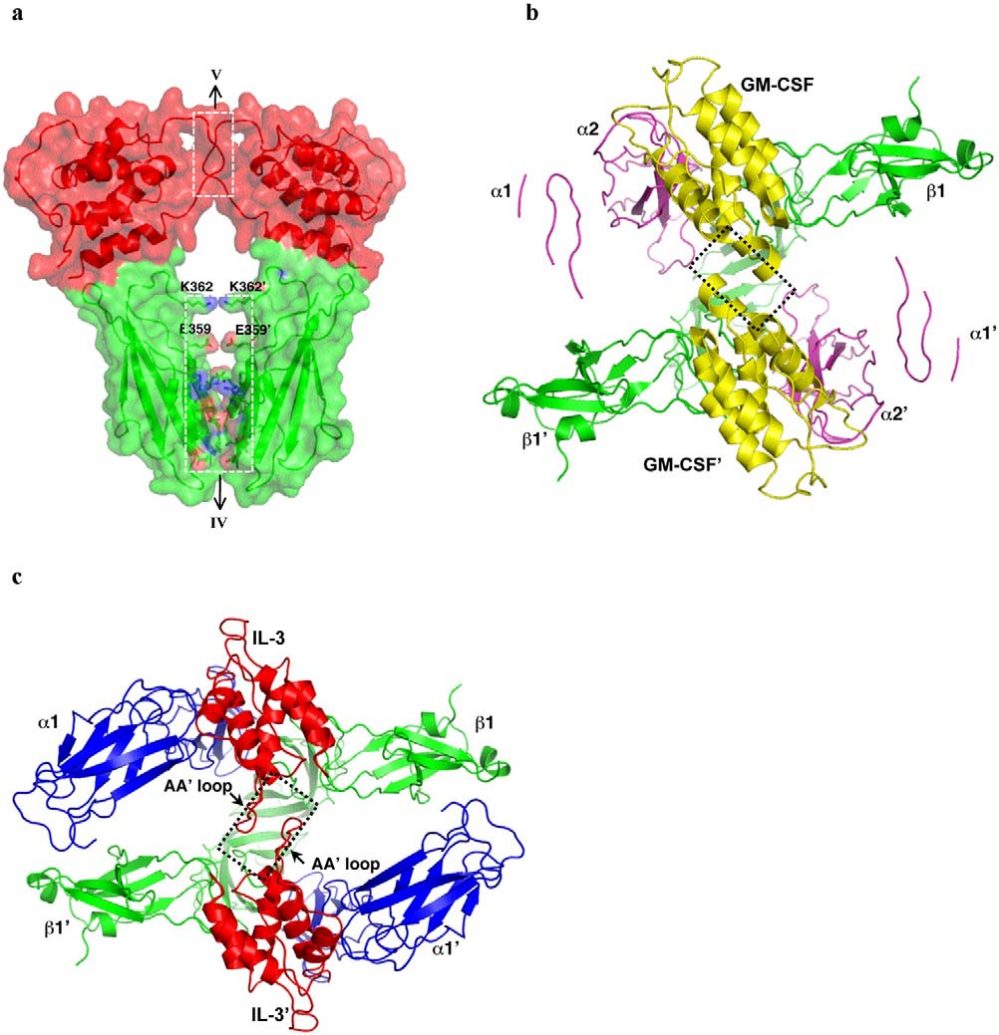
A cytokine–cytokine interaction interface in the dodecamer. (A) A ribbon/surface presentation of interface IV and V (dashed boxes) in the modeled IL-3:receptor complex. Only domain 4 of the interaction beta subunit and IL-3 are shown in this view. The two residues at interface IV (Glu359 and Lys362) that engage in potential charge repulsion are indicated. The arrows indicate the two fold symmetry axis that runs through both interface IV and V. (B) A top view of interface V in the crystal structure of the GM-CSF:receptor complex (dashed box) and its relative position with respect to other components of the complex. The receptor alpha subunit domain 1 showed only partial density of a few beta strands that are represented by backbone trace here. (C) A top view of interface V (dashed box) mediated by the AA′ loop in the modeled IL-3:receptor complex.

We applied symmetry operations derived from the GM-CSF:receptor crystal to generate both hexamer and dodecamer of the hIL-3:receptor complex (Figure 2A and 2B). The resulting higher-order complex shows that the components of the dodecamer fit nicely with each other. A unique feature of the receptors for GM-CSF, IL-3 and IL-5 is that the beta subunit forms an intertwined dimer [39]. In vitro binding studies suggest that domain 1 and domain 4 from different beta subunits act together to bind the cytokine ligand [40], [41]. The crystal structure of the GM-CSF:receptor complex reveals that this is indeed the case. Domain 1 of one beta subunit and domain 4 of the other form a composite surface to bind GM-CSF at interface II, such that two cytokines and two GMRα chains are cross linked by the beta dimer so as to form a 2∶2∶2 hexamer (Figure 1 in Hansen et al.[34]). In the crystal of the GM-CSF:receptor complex, two hexamers pack head-to-head together to form a dodecamer wherein domain 4 of two adjacent beta subunits contact each other through an extensive protein interface (Figure 3 in Hansen et al. [34]). This interface, referred to as interface IV, has been shown by mutagenesis and neutralizing antibodies to be important for receptor activation. The same interface is also present in the modeled IL-3:receptor complex (Figure 3A). As discussed below, we identified another protein-protein interaction interface involved in the assembly of the GM-CSF:receptor dodecamer (Figure 3B). This interface, termed interface V, is mediated by the loop region between helix A and helix B of GM-CSF bound to two different hexamers. This region of GM-CSF contains a short alpha helix that forms the major part of the interface. In the modeled hIL-3:receptor complex, the corresponding region of IL-3, the AA′ loop, is similarly positioned to form an analogous interaction interface in the dodecamer (Figure 3A and 3C).

### Comparing the IL-3:receptor structure model with biochemical data

In the following sections, we will analyze the modeled structure in the context of biochemical data to gain insights into the molecular details of IL-3:receptor interactions. We also seek to evaluate the dodecamer model of receptor activation through structure-function analysis of the higher-order IL-3:receptor complex.

### Protein interfaces involved in IL-3 binding

IL-3 binds the IL-3R alpha subunit with modest affinity through interface I but not to the beta subunit alone. Upon IL-3 binding to the alpha subunit, the beta subunit dimer is recruited to the complex through interfaces II and III (Figure 1A and 1B). These interfaces act cooperatively to stabilize the overall complex, resulting in high affinity binding of the cytokine ligand. We will discuss interface II first because it is constructed with minimal model manipulation. This interface is defined by the common beta subunit and by the experimentally derived IL-3 structure superimposed on GM-CSF as a rigid body. At interfaces I and III, on the other hand, loops of IL-3R alpha subunits required local adjustments to dock onto IL-3 and the beta subunit.

**Figure 4:**
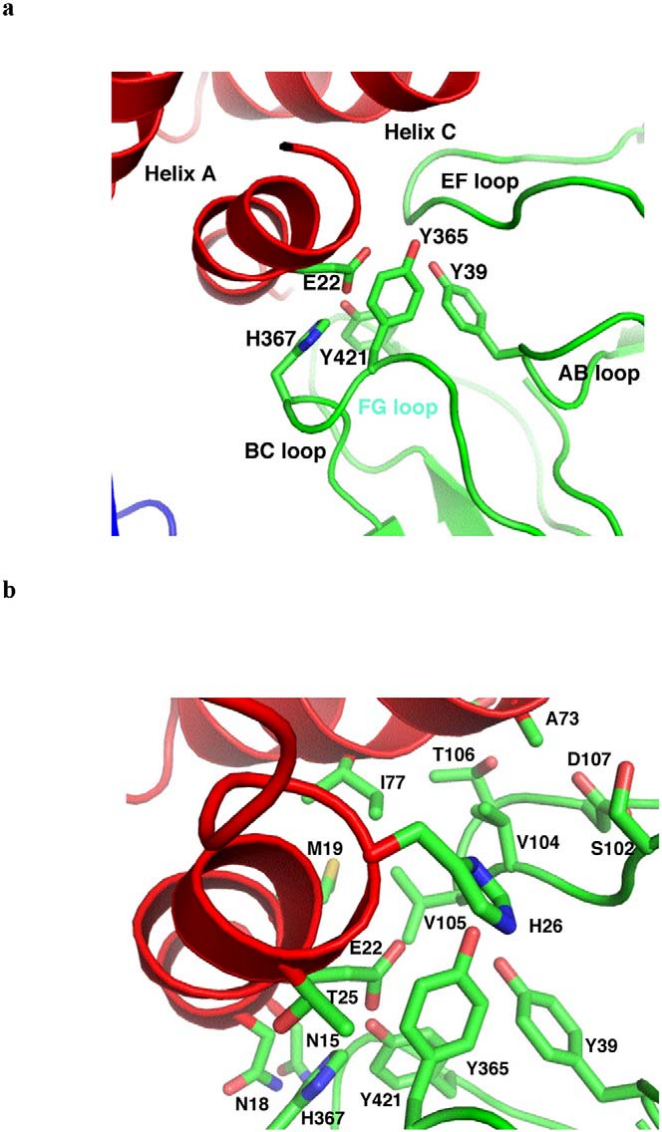
Protein–protein interactions at interface II. (A) A zoom-out view of the interface showing the secondary structural elements of IL-3 and the receptor involved in the reaction. The interaction between *Glu22* of IL-3 and a number of aromatic residues from the receptor beta subunit (Tyr39, Tyr365, His367, Tyr421) is shown. (B) A zoom-in view showing additional details of interface II, including the interactions between *Met19*, *Ile77* and *Ala73* of IL-3 and Ser102, Val104, Val105 and Thr106 on the receptor beta subunit.

At interface II (Figure 4), helix A and C of IL-3 bind a composite protein surface formed by the AB and EF loops of domain 1 of one beta subunit and the BC and FG loops of domain 4 of the other. A prominent feature of interface II is the intimate interaction between *Glu22* (residues in IL-3 are italicized throughout the text) of IL-3 and a group of aromatic residues of the beta subunit, including Tyr39, Tyr365, His367 and Tyr421 (Figure 4A). In addition to extensive van der Waals contacts, the carboxylate group of *Glu22* is in position to form a hydrogen bond with the phenolic hydroxyl group of Tyr421. An adjacent patch of interactions involves *Met19*, *Ser76*, *Ile77* and *Ala73* of IL-3 that pack against Ser102, Val104, Val105 and Thr106 on the beta subunit. *Asn15*, *Asn18*, *Thr25*, and *His26* of IL-3 are also in close proximity to contact the beta subunit (Figure 4B). The detailed interactions at interface II are similar to those seen in the GM-CSF:receptor complex but also show some differences. For example, a salt bridge between Lys72 of GM-CSF and Asp107 of the beta subunit is missing at interface II of the IL-3:receptor complex, whereas *Ile77* of IL-3 may bind the hydrophobic pocket formed by Ser102, Val104, Val105 and Thr106 of the beta subunit better than the proline counterpart (Pro76) in GM-CSF. The structural features of interface II are consistent with mutagenesis data on the functional roles of interface residues (Figure 1C). For example, the tight packing interaction at the center of the interface is highly sensitive to mutations of *Glu22* of IL-3 and Tyr421 of the beta subunit [30], [31], [33], [42], [43]. Even subtle changes, such as substituting Tyr421 with phenylalanine in the beta subunit, abolish the high affinity binding of IL-3 and GM-CSF to the receptor.

**Figure 5:**
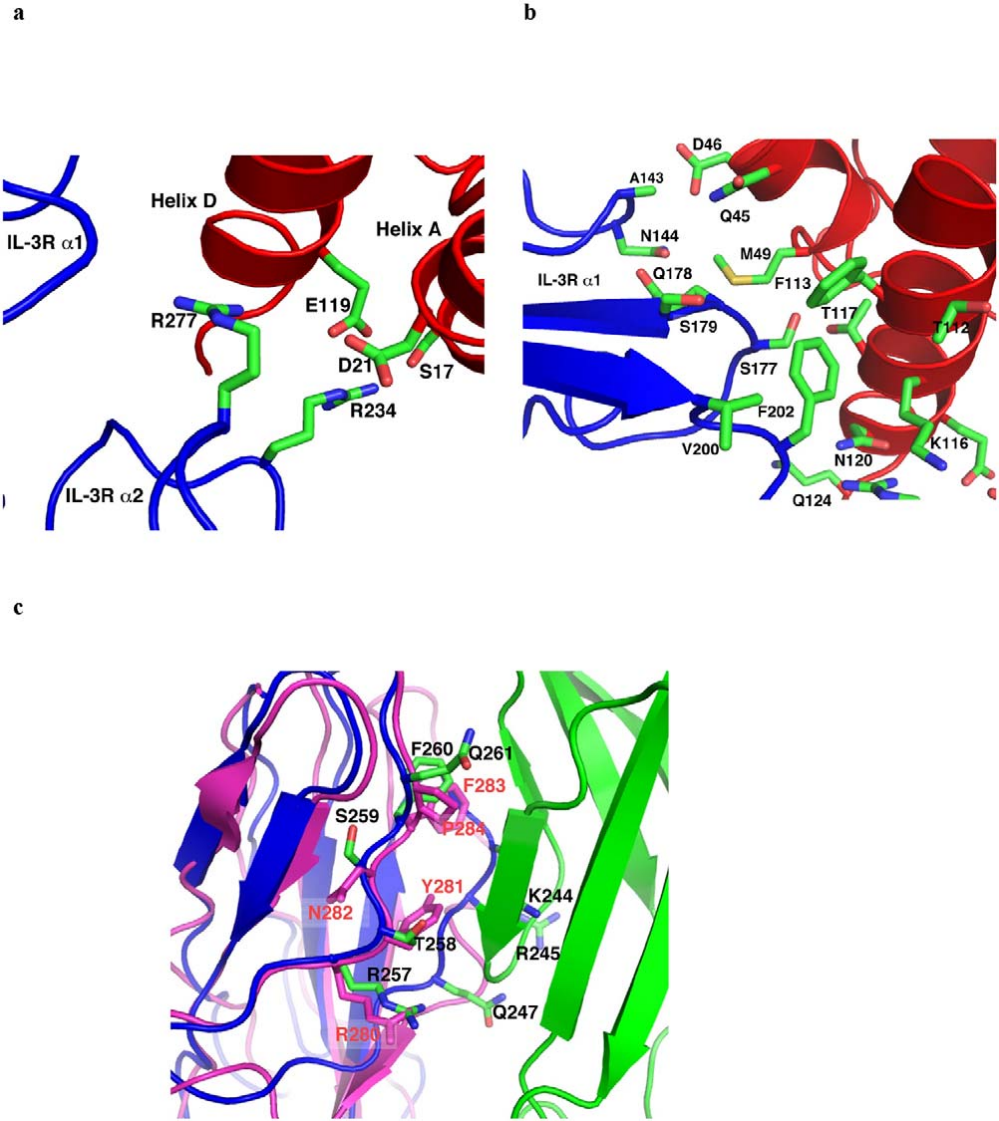
Protein–protein interactions at interfaces I and III. (A) A charged interaction patch between helix A of IL-3 and domain 2 of IL-3R alpha. Here *Asp21* and *Glu119* of IL-3 engage in electrostatic interaction with Arg277 and Arg234 of IL-3R alpha, respectively. (B) A hydrophobic interaction patch between IL-3 and IL-3R alpha domain 1. Note that Phe202 of IL-3R alpha inserts into a hydrophobic pocket formed by *Phe113*, *Thr112* and the aliphatic side chain of *Lys116* on IL-3. (C) Foreground: the loop region between Arg257 and Gln261 of IL-3R alpha (blue) is very similar to the corresponding region of GMR alpha (magenta) in sequence, structure and interaction with beta-subunit (green). Background: residues Lys244, Arg245 and Gln247 of IL-3R alpha also contact the beta subunit.

At interface I, helix A, A′ and D of IL-3 bind domain 1 and domain 2 of IL-3R alpha through two distinct patches of protein-protein interactions, one dominated by electrostatic interactions while the other rich in hydrophobic/van der Waals contacts. The charge interaction patch is formed between helix A of IL-3 and domain 2 of IL-3R alpha, where *Asp21* and *Glu119* of IL-3 are in position to engage in electrostatic interaction with Arg277 and Arg234 of IL-3R alpha, respectively (Figure 5A). Arg234 of IL-3R alpha is also in position to interact with *Ser17* of IL-3 through hydrogen bonding. For the hydrophobic patch, residues located on various loops of IL-3R alpha domain 1, including Val200, Phe202, Ser177–Ser179, and Ala143-Arg146, project toward the cytokine to interact with *Gln124*, *Asn120*, *Thr117*, *Lys116*, *Phe113*, *Gly42*, *Gln45*, *Asp46*, and *Met49* on helix D and A′ of IL-3. Most notably, Phe202 of IL-3R alpha is poised to insert into a hydrophobic pocket formed by *Phe113*, *Thr112* and the aliphatic side chain of *Lys116* on IL-3 (Figure 5B). Consistent with the structure model, a number of IL-3 residues located at interface I, including *Ser17*, *Asn18*, *Asp21*, *Arg108*, *Phe113*, *Lys116* and *Glu119* have been shown to be important for receptor binding and activation by mutagenesis [29], [30], [32] (Figure 1C). The loss of function for mutations *Ser17Lys*, *Asn18Lys*, *Thr25Arg* and *Glu119Arg* of IL-3 could be explained by charge repulsion with Arg234 and Arg277 of IL-3R alpha [32], while that for *Phe113A* could be explained by diminished hydrophobic interactions with Phe202 of IL-3R alpha. Interestingly, a number of IL-3 mutations, such as *Lys116Val*, *Gln45Val*, *Lys116Trp*, and *Thr112Arg*, showed enhancement in receptor binding and/or cellular activities [29], [31], [32]. Remarkably, these residues are located around *Phe113* in the folded structure of IL-3. Substitution of these hydrophilic residues with hydrophobic residues or residues with longer aliphatic side chain could therefore augment the hydrophobic interaction at interface I and hence enhance the activity of mutated IL-3.

At interface III, the loop region of Arg257-Gln261 in IL-3R alpha, which forms a major part of the binding site of beta domain 4, is very similar to the corresponding region of GMR alpha in sequence, structure and interaction with beta-subunit (Figure 5C). On the other side of the beta sandwich (Figure 5C), residues Lys244, Arg245 and Gln247 of IL-3R alpha are also involved in beta subunit binding. This part of interface III in the IL-3:receptor complex is different from that of the GM-CSF:receptor complex because the loop region following the β-strand Lys235-Leu240 is much shorter in IL-3Rα than in GMRα (Figure S5A). Overall, interface III in the IL-3:receptor and GM-CSF:receptor complexes show significant similarity as well as some differences.

### Protein–protein interactions involved in the assembly of the dodecamer

A dodecamer complex is formed in the crystal of the GM-CSF:receptor complex [34]. Interactions between domain 4 of two adjacent beta subunits belonging to two different hexamers constitute a significant part of the protein interface (interface IV) in the assembly of the dodecamer. Residues on several beta strands of domain 4, including Gln346 - Asp352, Arg357, Glu359, Lys362 and Arg432-Asp435, are in position to contact each other at this interface [34]. These interactions are also conserved in the IL-3:receptor dodecamer complex (Figure 3A), which may also be involved in receptor activation. Consistent with this hypothesis, extensive mutations at interface IV not only reduced the activity of the GM-CSF:receptor complex but also that of the IL-3:receptor complex [34].

In the crystal structure of the GM-CSF:receptor complex, Asn261 of the α subunit domain 2 of one hexamer makes a small contact to Glu430 and Arg 432 of the beta subunit domain 4 of the other hexamer (Figure S6). The contribution of this interaction to the dodecamer assembly is likely small due to the limited contact. Moreover, it is absent in the modeled hIL-3:receptor dodecamer complex because IL-3R alpha has a much shorter loop in this region than GMR alpha (Figure S5A). These observations raise the question on how cytokine controls the assembly of the dodecamer.

**Figure 6:**
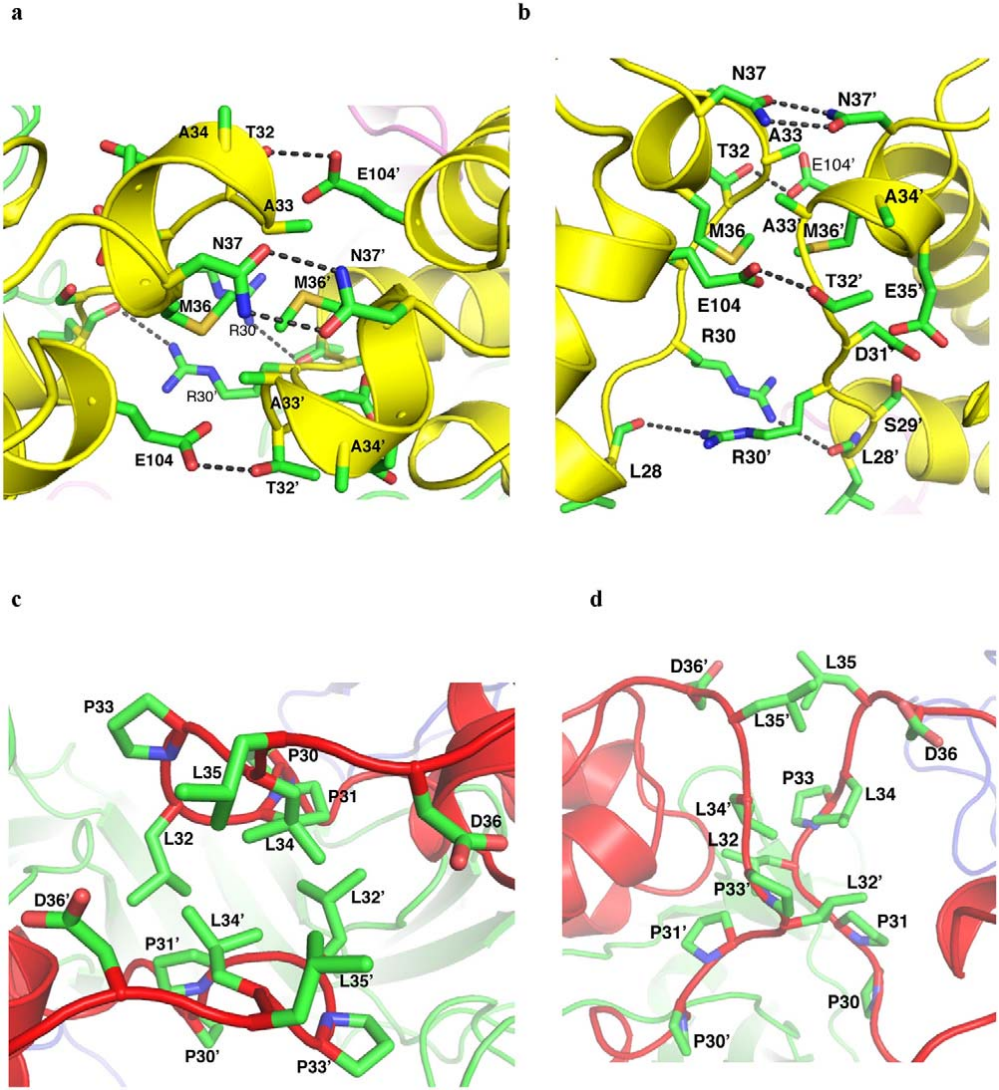
Detailed protein–protein interactions at interfaces V. (A) Detailed GM-CSF:GM-CSF interactions in the crystal structure of the GM-CSF:receptor dodecamer (top view); (B) side view of (A); (C) Detailed IL-3:IL-3 interactions in the modeled structure of the IL-3:receptor dodecamer (top view); (D) side view of (C).

Further examination of the GM-CSF:receptor crystal reveals that another protein-protein interaction interface, mediated by the AB loop of GM-CSF, forms an integral part of the dodecamer (Figure 3B, Figure 6A and 6B). This interface, termed interface V, is located directly above interface IV and shares the same twofold symmetry axis with interface IV (Figure 3A). These structural features suggest that interface V and IV may form concertedly and act cooperatively in the assembly of the dodecamer. Interface V in the GM-CSF:receptor complex buries about 646 Å^2^ solvent accessible surface area that includes residues *Leu28-Asn37* and *Glu104* from two interacting GM-CSF molecules. The interface is mediated by two short helices and has a hydrophobic core consisting of *Ala33*, *Met36*, *Ala33′* and *Met36′* (the prime sign denotes residues from symmetry partner), which is surrounded by extensive van der Waals contacts and hydrogen bond interactions (Figure 6A). For example, *Arg30* forms a hydrogen bond with the main chain carbonyl of *Leu28′*, *Thr32* forms a hydrogen bond with *Glu104′*, while *Asn37* form a bidendate hydrogen bond with *Asn37′* (Figure 6B). These structural features suggest that interface V has excellent shape and chemical complementarity and likely makes significant contribution to the dodecamer assembly. It is interesting to note that interface V in GM-CSF is bounded by two glycosylation sites, Asn27 and Asn37, suggesting that the sugar chain attached at the periphery of interface V could augment the protein-protein interaction and hence the dodecamer stability [44], [45].

In the modeled IL-3:receptor complex, a similar interface is formed between the AA′ loop of IL-3 bound to two opposing hexamers (Figure 3A and 3C). Both IL-3 and GM-CSF have an extended loop between helix A and B but share little sequence conservation in this region (Figure 1C), suggesting that interface V can form in the two family members but not between them. The AA′ loop of IL-3 contains a characteristic PPLPLL motif (residues 30–35 in human IL-3). The exposed and hydrophobic nature of this motif suggests that the AA′ loop has a high tendency to bind other hydrophobic protein surface. Here in the IL-3:receptor dodecamer complex, the PPLPLL motifs from two opposing IL-3 molecules are poised to interact with each other (Figure 6C and 6D). Although the detailed interactions at interface V of IL-3 await direct experimental determination, our modeling suggest that *Leu32*, *Leu34* and *Leu35* are in positions to interact with their counterparts in the symmetric partner, much like the leucine zipper interaction observed in many protein complexes. However, unlike classical leucine zippers where leucine residues are presented by alpha helix, the conformation of the AA′ loop of IL-3 seems to be partially restricted by a series of proline residues, which may be required to reduce non-specific hydrophobic interactions.

**Figure 7:**
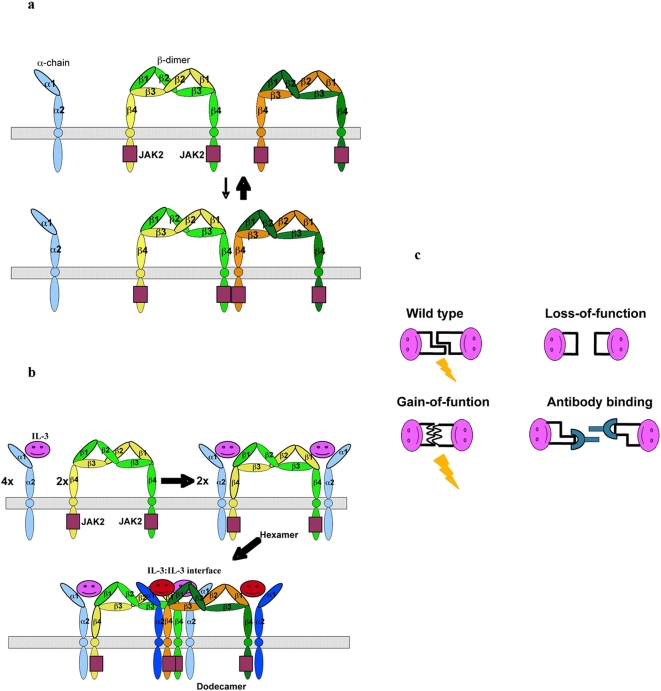
Activation mechanism of the IL-3:receptor complex. (A) In the absence of cytokine, the receptor subunits are scattered on the cell surface, where the beta dimer, which binds JAK2 through its cytoplasmic region, form transient tetramer through interface IV that may contribute to residual phosphoryaltion of JAK2. (B) In the presence of IL-3, the cytokine and receptor subunits assembly into a dodecamer that is stabilized by IL-3:IL-3 interaction, leading to robust signal activation through JAK2 trans-phosphorylation. (C) A variety of experimental data showed that modifications of the AA′ loop of IL-3, including deletion, loss-of-function mutations, and antibody binding, diminished the activity IL-3, whereas gain-of-function mutations are also frequently observed in this region that enhanced the IL-3 without affecting the receptor binding affinity.

Though far away from the receptor binding sites, the PPLPLL motif (*Pro30*-*Leu35*) has been shown to be a functionally important site on IL-3 by a number of experiments. In mapping functional sites of IL-3 using monoclonal antibodies that bind IL-3 and inhibit its activity, Lokker *et al.* showed that the epitopes of two neutralizing antibodies were located between *Leu32* and *Asp36* [26], [35]. Deletion of a significant portion of this region, such as *Pro30-Leu33 (del-Pro30-Leu33)* and *Pro30-Leu34 (del-Pro30-Leu34)*, diminished the activity of IL-3. Site-specific mutations of residues in this region affected the activity of IL-3 both positively and negatively. While the *Leu34Glu* mutation showed little effect on the function of IL-3, the *Leu34Gly* mutant showed much reduced activity than the wild type IL-3. On the other hand, substitution of *Pro33* with Asn (*Pro33Asn*) and Gly (*Pro33Gly*) enhanced the specific activity of IL-3. Gain-of-function mutations of IL-3 have also been identified in the PPLPLL motif in a saturation mutagenesis study [31], where *Leu32Arg*, *Leu34Ser* and *Leu34Met* showed increased activity. Many of the loss-of-function mutations such as *del-Pro30-Leu33*, *del-Pro30-Leu34*, and *Leu34Gly* also showed decreased affinity for the receptor [35]. How these modifications affect the binding of IL-3 to its receptor is not clear since the AA′ loop does not make direct contact to either subunits of the receptor in the modeled structure (Figure 3A and 3C). While these negative results seem to contradict the model of interface V mediated signal activation through dodecamer assembly, it must be kept in mind that loss-of-function of mutations often involve complex but trivial mechanisms, such as protein mis-folding or aggregation, especially considering the hydrophobic nature of the AA′ loop (see discussion below). By contrast, the gain-of-function mutations can often be much more mechanistically revealing. Here these mutations enhanced the specific activity of IL-3 substantially with little to modest effect on the binding affinity for the receptor. Most notably, the *Pro33Gly* mutant showed 14-fold increase in activity but virtually the same receptor affinity as compared with the wild type protein in cell-based assays, indicating that modifications in the PPLPLL motif of the AA′ loop can affect the function of IL-3 through mechanisms independent of receptor binding. Animal studies of an IL-3 triple mutant (*Pro33Gly/Trp104Glu/Asn105Asp*) also showed that modifications of the PPLPLL motif and its nearby regions could enhance the specific activity of IL-3 significantly under physiological conditions (Kunmei Ji and Zhigang Liu, data not shown). Mutations on interface IV have also been shown to disrupt the function of the receptor without affecting the binding of the cytokine [34]. These observations suggest that interface V identified here contribute to the activation of the receptor through a similar mechanism to interface IV, namely the assembly of the dodecameric cytokine:receptor complex (Figure 7).

## Discussion

The homology model of the IL-3:receptor complex generated in this study provides a molecular framework to analyze a large body of mutagenesis data on the roles of specific residues in IL-3:receptor complex formation and function. These analyses reveal three prominent features at the IL-3:receptor interface (Figures 1, 4, and 5). First is the tight interaction between *Glu22* of IL-3 and a group of aromatic residues on the receptor beta subunit. This patch of interaction is highly sensitive to mutations, suggesting its critical role in the binding of IL-3 to the receptor [30], [31], [33], [42], [43]. The second feature is a perfectly matched charge interface between *Asp21* and *Glu119* of IL-3 and Arg277 and Arg234 of IL-3R alpha. This feature is consistent with a series of mutational studies showing that *Ser17Lys*, *Asn18Lys*, *Thr25Arg* and *Glu119Arg* can severely disrupt the function of IL-3, presumably by introducing charge repulsion with Arg277 and Arg234 of IL-3R alpha [29], [30], [32]. The third feature is a hydrophobic interface formed between Phe202 of IL-3R alpha and a hydrophobic pocket lined by *Phe113*, *Thr112* and the aliphatic side chain of *Lys116* on IL-3. This feature provides potential interpretations for the loss-of-function mutation of *Phe113Ala* as well as the gain of function mutations of *Lys116Val*, *Gln45Val*, *Lys116Trp*, and *Thr112Arg*, which may decrease or increase the hydrophobic interaction, respectively [29], [31], [32].

A large number of IL-3 residues, including some at the IL-3:receptor interface, show high tolerance to mutations [31], [33]. This fact does not necessarily indicate poor quality of the structure of the IL-3:receptor complex generated by homology modeling. It is very common that interactions observed in experimentally derived structures may or may not have energetic contribution to binding and function. As shown in this study, structure-based analysis of specific mutations that alter the binding and function with defined chemical changes is needed to understand the functional mechanism of protein-protein interactions between IL-3 and its receptor. Overall, the homology model of the IL-3:receptor complex is in excellent agreement with previously published mutagenesis data, thus providing a reliable framework for analyzing the IL-3:receptor interaction.

The beta subunit of IL-3, GM-CSF, and IL-5 receptors forms a stable dimer [34], [39], where a cytokine-binding site is located at each end of the extended dimer structure. Such an unusual structural feature leads to the assembly of the cytokine:receptor hexamer when two cytokine:receptor alpha subunit complexes are recruited to the beta dimer (Figures 2 and 7). Although the cytoplasmic domain of the receptor alpha chain is required for function [13], [14], it does not appear to be associated with any signaling kinases [15]–[17], [34]. The cytoplasmic region C-terminal to domain 4 of the beta subunit, on the other hand, has been shown to bind JAK2 that is capable of initiating the signaling cascade [34]. However, the membrane proximal domain (domain 4) of the beta subunit in the 2∶2∶2: hexamer is 120 Å apart, raising the question on how trans-phosporyaltion of JAK2 and signal activation are achieved in the cytokine:receptor complex.

The crystal structure of the GM-CSF:receptor complex provides a potential answer to this question. In the crystal lattice, two GM-CSF:receptor hexamers stack against each other to form a dodecamer, which brings domain 4 of two beta subunits from different hexamers in close proximity. Initial support for this model of dodecamer-dependent activation came from mutagenesis analysis of a protein interface (interface IV) between domain 4 of two adjacent beta subunits (Figure 3A). However, a puzzling aspect of this model is how the assembly of the dodecamer, which is apparently required for receptor activation, is coupled to cytokine binding to the receptor, which must be the initiation event of signaling. Interface IV is mediated entirely by the beta subunit, suggesting that it could form stochastically and transiently without the binding of cytokine. In this regard it is interesting to note that low level phosphorylation of JAK2 does occur in the absence of cytokine (see Figure 4C in Hansen et al. [34]) (Figure 7). The protein-protein interactions at interface IV are largely polar without significant chemical complementarity in hydrogen bonding and electrostatic interaction. In fact, a number of charged residues such as Glu359 and Lys362 seem to engage in electrostatic repulsion (Figure 3A), which may play a role in reducing background signaling. These structural features suggest that interface IV will not form stably in solution by itself. However, its structural integrity is still important for function as extensive but not limited mutations at this interface diminished the activity of the receptor. In the event of cytokine-mediated signaling, interface IV will most likely work with other protein interaction interfaces that provide the driving force for the dodecamer assembly and signaling activation.

One such interface is a cytokine-cytokine interaction identified in this study, which we termed interface V. In the crystal structure of the GM-CSF:receptor complex, the AB loop of GM-CSF bound to adjacent hexamers contact each other through a significant binding interface (Figure 3B, Figure 6A and 6B). Although no functional data is currently available for the AB loop of GM-CSF, this interface could contribute to the dodecamer assembly and receptor activation. In the modeled IL-3:receptor complex, the AA′ loop of two adjacent IL-3 molecules are similarly positioned to form an interaction interface through the PPLPLL motif (Figure 3C, Figure 6C and 6D). Here a large body of data is available to assess the functional roles of this motif in IL-3. While the importance of the PPLPLL motif in IL-3 has long been demonstrated by neutralizing antibodies and by mutagenesis [26], [35], the observation that a number of mutations in this region can increase the specific activity of IL-3 without changing the binding affinity of the receptor has been puzzling [35]. In the dodecamer of the GM-CSF:receptor complex, mutations at interface IV have also been shown to disrupt the function of the receptor without changing the binding of IL-3 and GM-CSF [34]. These observations suggest that interface V, which is mediated entirely by cytokine-cytokine interactions, could contribute to the receptor activation through the assembly of the dodecamer (Figure 7).

The AA′ loop of IL-3 is conserved between human, chimapanzee and gibbon but shows variations across a range of other species including rhesus, tamarin, marmoset, sheep, bovin, mouse and rat. This region of IL-3 is also tolerant to a series of mutations [31], suggesting that sequence conservation in this region is not absolutely required for maintaining interface V interactions. Because the AA′ loop makes few contacts to the main body of the IL-3 structure, it is highly exposed and flexible. Interactions between the AA′ loop from two adjacent IL-3 molecules most likely involve co-folding. Such a mechanism of interaction will make interface V more tolerant to mutations than protein-protein interaction mediated by rigid structures, as long as the mutations, which will occur simultaneously on both sides of the interface, permit the co-folding of the modified AA′ loop. This mechanism of interaction may also explain why a large number of gain-of-function mutations could be generated by modifying the AA′ loop (Figure 1C and Figure 7C). In the context of natural cytokine:receptor complex, sequence divergence and the co-folding mechanism at interface V would provide a way to confer specie-specific activity of IL-3 or member-specific function in the IL-3, GM-CSF, IL-5 family. In this regard, it is interesting to note that IL-5 forms a stable dimer [46]. Although the IL-5 dimer interface is incompatible with interface IV observed in the GM-CSF:receptor dodecamer [34], it may represent a cytokine-cytokine interaction that has evolved into a stable form for function, probably through the assembly of a different type of higher-order cytokine:receptor complex.

In summary, our analysis of the IL-3:receptor complex by homology modeling and published mutagenesis data provides further evidence for the dodecamer-model of receptor activation proposed for GM-CSF and IL-3 [34]. Our studies suggest that the AA′ loop in IL-3 and the analogous region in GM-CSF participate in the assembly of the dodecamer. Such a revised model of dodecamer-dependent activation solves the paradox in the previous model and provides a mechanism by which cytokines control the activation of receptor-mediated signaling (Figure 7).

## Supporting Information

Figure S1 Sequence alignment between domain 1 of IL-3 receptor alpha chain (IL3R-α1) and the corresponding region in IL-13 (IL13R-α). The arrows above the sequence denote regions of beta strand. Identity and similarity are indicated by standard conventions below the sequence. These conventions are used throughout the illustration. The sequence between residue 176 and 177 in IL-3 corresponds a large loop insertion in IL-13R alpha chain (residues192–199).
(1.56 MB TIF)

Figure S2 Sequence alignment between domain 2 of IL-3 receptor alpha chain (IL3R-α2) and the corresponding region in GM-CSF receptor alpha chain (GMR-α2).
(1.56 MB TIF)

Figure S3 Sequence alignment between the full length IL-3 receptor alpha chain (IL3R-α) and IL-13 receptor alpha chain (IL13R-α).
(1.56 MB TIF)

Figure S4 The structure of IL-3 (red) is superimposed on that of GM-CSF (yellow) as a rigid body. Representative residues involved in the core packing of the helix bundle are shown to demonstrate the similar folding interactions between the two proteins. The residues are colored according to their host proteins. Only residues of IL-3 are labeled. The AA′ loop of IL-3 and the AB loop of GM-CSF are indicated.
(1.56 MB TIF)

Figure S5 (A) Superposition of domain 2 of IL-3 receptor alpha chain (IL-3R-α2, blue) with the corresponding domain of GM-CSF receptor alpha chain (GMR-α2, magenta). Representative residues involved in the packing of the beta sandwich core are shown to demonstrate the similar folding interactions between the two proteins. The residues are colored according to their host proteins. Only residues of IL-3 are labeled. The two long loops in GMR-α2 located at the end of the beta barrel correspond to much shorter loops in IL-3. (B) Superposition of domain 1 of IL-3 receptor alpha chain (IL-3R-α1, blue) with the corresponding domain of IL13 receptor alpha chain (IL13Rα, pink). Representative residues involved in the packing of the beta sandwich core are shown to demonstrate the similar folding interactions between the two proteins. The residues are colored according to their host proteins. Only residues of IL-3 are labeled.
(1.55 MB TIF)

Figure S6 In the crystal structure of the GM-CSF:receptor complex, Asn261 of the α subunit domain 2 of one hexamer makes a small contact to Glu430 and Arg 432 of the beta subunit domain 4 of the other hexamer.
(1.56 MB TIF)
