# Supplementary figures and images for: A Cytokine–Cytokine Interaction in the Assembly of Higher-Order Structure and Activation of the Interleukine-3:Receptor Complex
Source: PLoS One. 2009 Apr 7;4(4):e5188. doi: 10.1371/journal.pone.0005188 (PMC2662821; doi:10.1371/journal.pone.0005188)

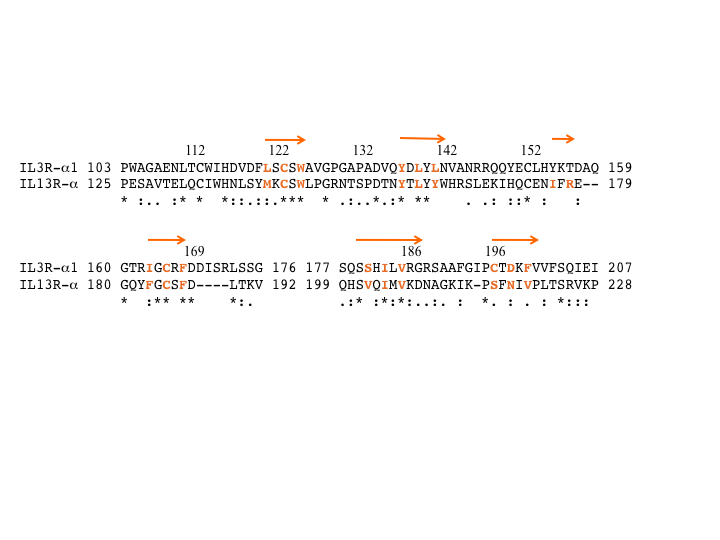

Supplement: Figure S1 — Sequence alignment between domain 1 of IL-3 receptor alpha chain (IL3R-α1) and the corresponding region in IL-13 (IL13R-α). The arrows above the sequence denote regions of beta strand. Identity and similarity are indicated by standard conventions below the sequence. These conventions are used throughout the illustration. The sequence between residue 176 and 177 in IL-3 corresponds a large loop insertion in IL-13R alpha chain (residues192–199). (1.56 MB TIF) [file pone.0005188.s001.tif]

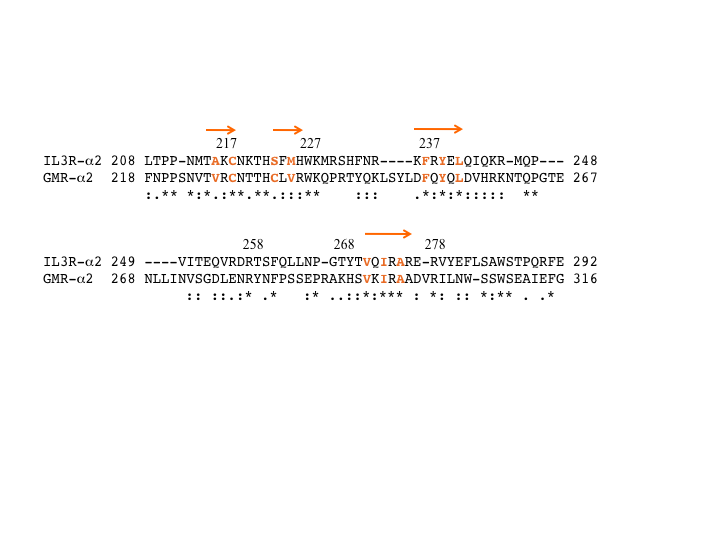

Supplement: Figure S2 — Sequence alignment between domain 2 of IL-3 receptor alpha chain (IL3R-α2) and the corresponding region in GM-CSF receptor alpha chain (GMR-α2). (1.56 MB TIF) [file pone.0005188.s002.tif]

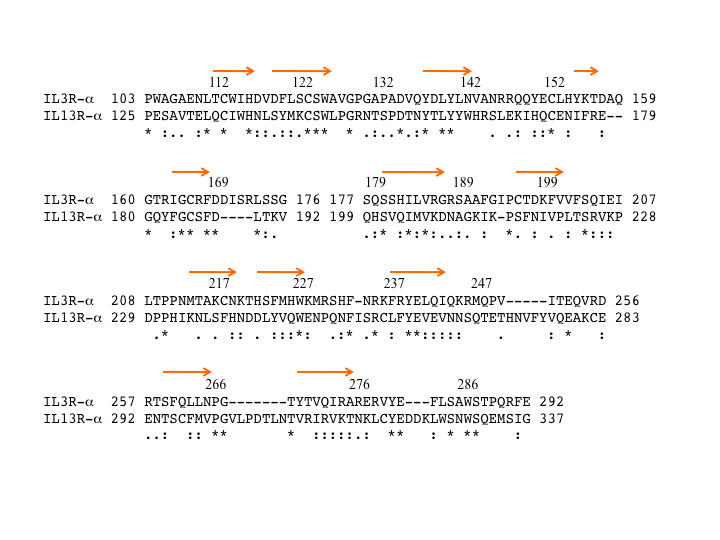

Supplement: Figure S3 — Sequence alignment between the full length IL-3 receptor alpha chain (IL3R-α) and IL-13 receptor alpha chain (IL13R-α). (1.56 MB TIF) [file pone.0005188.s003.tif]

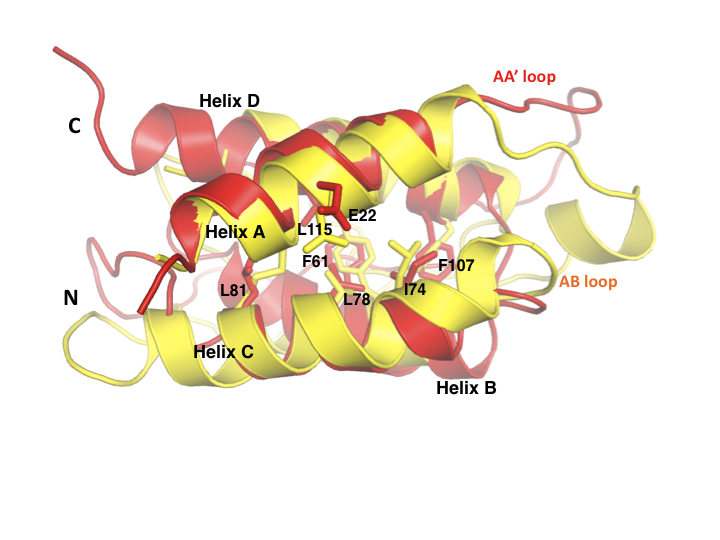

Supplement: Figure S4 — The structure of IL-3 (red) is superimposed on that of GM-CSF (yellow) as a rigid body. Representative residues involved in the core packing of the helix bundle are shown to demonstrate the similar folding interactions between the two proteins. The residues are colored according to their host proteins. Only residues of IL-3 are labeled. The AA′ loop of IL-3 and the AB loop of GM-CSF are indicated. (1.56 MB TIF) [file pone.0005188.s004.tif]

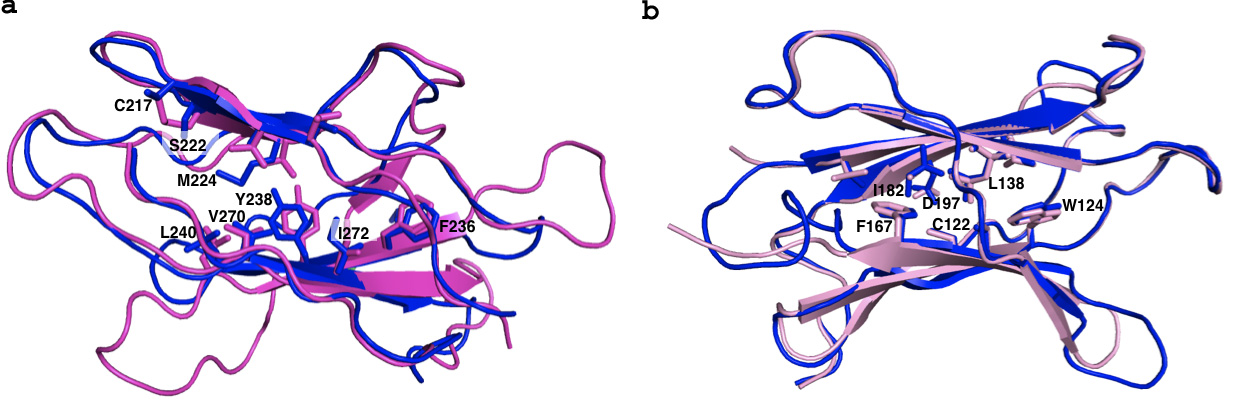

Supplement: Figure S5 — (A) Superposition of domain 2 of IL-3 receptor alpha chain (IL-3R-α2, blue) with the corresponding domain of GM-CSF receptor alpha chain (GMR-α2, magenta). Representative residues involved in the packing of the beta sandwich core are shown to demonstrate the similar folding interactions between the two proteins. The residues are colored according to their host proteins. Only residues of IL-3 are labeled. The two long loops in GMR-α2 located at the end of the beta barrel correspond to much shorter loops in IL-3. (B) Superposition of domain 1 of IL-3 receptor alpha chain (IL-3R-α1, blue) with the corresponding domain of IL13 receptor alpha chain (IL13Rα, pink). Representative residues involved in the packing of the beta sandwich core are shown to demonstrate the similar folding interactions between the two proteins. The residues are colored according to their host proteins. Only residues of IL-3 are labeled. (1.55 MB TIF) [file pone.0005188.s005.tif]

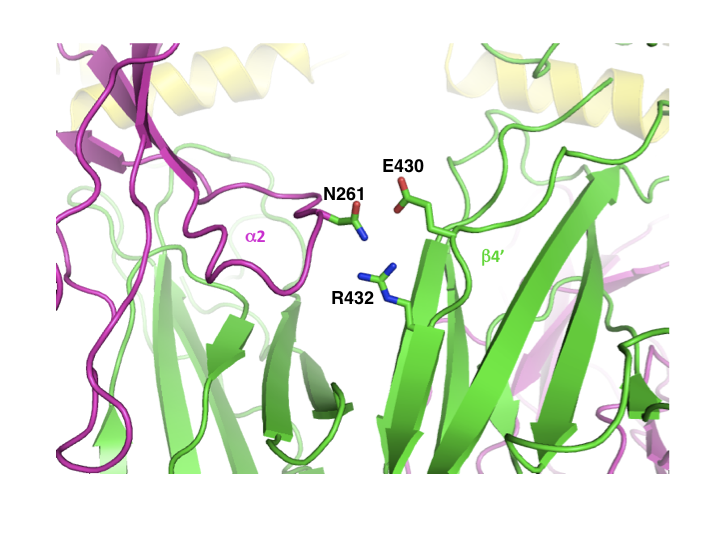

Supplement: Figure S6 — In the crystal structure of the GM-CSF:receptor complex, Asn261 of the α subunit domain 2 of one hexamer makes a small contact to Glu430 and Arg 432 of the beta subunit domain 4 of the other hexamer. (1.56 MB TIF) [file pone.0005188.s006.tif]
